# Comparison of effects of green tea catechins on apicomplexan hexose transporters and mammalian orthologues

**DOI:** 10.1016/j.molbiopara.2009.06.008

**Published:** 2009-11

**Authors:** Ksenija Slavic, Elvira T. Derbyshire, Richard J. Naftalin, Sanjeev Krishna, Henry M. Staines

**Affiliations:** a Centre for Infection, Division of Cellular and Molecular Medicine, St. George's, University of London, Cranmer Terrace, London SW17 0RE, UK; b Physiology, Franklin Wilkins Building, King's College London, Waterloo Campus, London SE1 9HN, UK

**Keywords:** BboHT1, *Babesia bovis* hexose transporter 1, EC, (−)-epicatechin, ECG, (−)-epicatechin-gallate, EGC, (−)-epigallocatechin, EGCG, (−)-epigallocatechin-gallate, GLUT1/5, mammalian facilitative glucose transporter 1/5, 3OMG, 3-*O*-methyl-d-glucose, PfHT, *Plasmodium falciparum* hexose transporter, Glucose, Transport, GLUT1/5, PfHT, BboHT1, Oocyte

## Abstract

Here we have investigated the inhibitory properties of green tea catechins on the *Plasmodium falciparum* hexose transporter (PfHT), the *Babesia bovis* hexose transporter 1 (BboHT1) and the mammalian facilitative glucose transporters, GLUT1 and GLUT5, expressed in *Xenopus laevis* oocytes. (−)-Epicatechin-gallate (ECG) and (−)-epigallocatechin-gallate (EGCG) inhibited d-glucose transport by GLUT1 and PfHT, and d-fructose transport by GLUT5, with apparent *K*_*i*_ values between 45 and 117 μM. BboHT1 was more potently inhibited by the ungallated catechins (−)-epicatechin (EC) and (−)-epigallocatechin (EGC), with apparent *K*_*i*_ values of 108 and 168 μM, respectively. Site-directed mutagenesis experiments provided little further support for previously reported models of catechin binding to hexose transporters. Furthermore, *P. falciparum* growth inhibition by catechins was not affected by the external d-glucose concentration. Our results provide new data on the inhibitory action of catechins against sugar transporters but were unable to elucidate the antimalarial mechanism of action of these agents.

The major polyphenol constituents of green tea (*Camellia sinensis*) are catechins, shown in Table 1 ((−)-epicatechin (EC), (−)-epicatechin-gallate (ECG), (−)-epigallocatechin (EGC) and (−)-epigallocatechin-gallate (EGCG)). Green tea catechins have potent antioxidant effects that have been investigated extensively, for example, as neuroprotective agents [1] and as chemopreventative/chemotherapeutic agents for cancers [2] due to inhibitory effects on angiogenesis [3], which in turn inhibit tumour growth and metastasis. Green tea catechins are also anti-infectious disease agents with antimicrobial [4] and antiviral activity [5].

Recently, green tea extract and its individual catechins have shown antimalarial activity against asexual blood-stage parasites [6,7]. In these studies, the gallated catechins, ECG and EGCG inhibited *Plasmodium falciparum* (strains NF54, K1 and 3D7) growth, with IC_50_ values (the concentration of inhibitor required to inhibit 50% of parasite growth) between 10 and 40 μM. The ungallated catechins were far less potent, with IC_50_ values in excess of 100–300 μM. Sannella et al. [6] were unable to determine a definitive mechanism of antimalarial action for catechins, although an antifolate mechanism of action was investigated and found to be unlikely. Tasdemir et al. [7] suggested fatty acid biosynthesis might be the target of gallated catechins but did not validate this.

Naftalin et al. [8] reported that gallated catechins are potent inhibitors of mammalian facilitative glucose transporter 1 (GLUT1)-mediated d-glucose transport in human erythrocytes, as sub-micromolar concentrations produce half maximal inhibitions when measuring zero-*trans* d-glucose efflux. Molecular modelling of GLUT1 also identified residues that may act as anchoring points for gallated catechins. These findings may also be relevant to the *P. falciparum* hexose transporter, PfHT, a parasite plasma membrane-localised protein that is the major route for parasite d-glucose and d-fructose uptake [9,10]. PfHT has been validated as a novel antimalarial drug target [11]. Here we hypothesised that the antimalarial activity of gallated catechins could be due to the inhibition of d-glucose uptake via PfHT.

The effect of the green tea catechins, EC, ECG, EGC and EGCG, on d-glucose transport via PfHT, GLUT1 and the *Babesia bovis* hexose transporter 1 (BboHT1; [12]) and d-fructose transport via GLUT5 was assayed in *Xenopus laevis* oocytes expressing each of the hexose transporters, using methods described previously [12]. The compounds were tested initially at a concentration of 0.5 mM (data not shown). In experiments performed at room temperature during the initial linear phase of uptake (10–20 min, depending on the expressed transporter), the transport of d-glucose (38 μM) via PfHT and GLUT1 and d-fructose (100 μM) via GLUT5 were inhibited to a significantly greater extent (*P* < 0.001; ANOVA with Tukey's post-test) by catechins containing a gallate group (i.e. ECG and EGCG) than by ungallated catechins (i.e. EC and EGC). In contrast, the opposite was observed for the effect of catechins on d-glucose uptake via BboHT1 (i.e. EC and EGC were significantly (*P* < 0.001; ANOVA with Tukey's post-test) more effective than ECG and EGCG). The two most effective catechins against each transporter were tested further over a range of concentrations. *K*_*i*_ values for each inhibitor were determined and presented in Table 1. *K*_*i*_ values for ECG and EGCG in the case of PfHT, GLUT1 and GLUT5 and *K*_*i*_ values for EC and EGC in the case of BboHT1 were similar (*P* = 0.13, 0.24, 0.96 and 0.42, respectively; unpaired, two-tailed, Student's *t*-test).

Given that d-glucose is metabolised after uptake into *X. laevis* oocytes, the effect of ECG was also tested on the uptake of 3-*O*-methyl-d-glucose (3OMG; a non-metabolised d-glucose analogue) via PfHT, which has similar *K*_m_ values [9] for the transport of both d-glucose (1.0 mM) and 3OMG (1.3 mM). The *K*_*i*_ value derived for the effect of ECG on the transport of 3OMG (17 μM) via PfHT was 18 ± 3 μM (mean ± SEM; *n* = 3). This was not significantly different from the *K*_*i*_ value derived for the effect of ECG on d-glucose transport via PfHT (*P* = 0.07; unpaired, two-tailed, Student's *t*-test). The relatively low *K*_*i*_ value for ECG inhibition of 3OMG transport clearly demonstrates that catechins inhibit sugar transport via PfHT rather than having an intracellular metabolic effect.

Our results show that the transport of d-glucose via GLUT1 is more susceptible to inhibition by gallated than ungallated catechins, consistent with the findings of Naftalin et al. [8]. However, the *K*_*i*_ values for the effect of gallated catechins on d-glucose transport via GLUT1 presented here are two orders of magnitude higher than those published previously (45 versus 0.14 μM for ECG and 89 versus 0.97 μM for EGCG, respectively). There may be many reasons for these differences but they are most likely to be due to (i) the different microenvironments of erythrocytes compared with *X. laevis* oocytes, resulting in different ligand activities at the membrane surface and/or (ii) the method of measuring transport (zero trans efflux versus influx).

Furthermore, d-glucose transport by PfHT and d-fructose transport by GLUT5 are blocked by gallated catechins with similar kinetic constants to those reported here for d-glucose transport via GLUT1. This suggests that gallated catechins may well interact with each of these diverse hexose transporters in a similar manner. A contrasting observation though, is that d-glucose transport by BboHT1 is more susceptible to ungallated catechins. This reversed pharmacological profile has not been observed for any other hexose transporter or, in general, other processes that are targets for catechins (e.g. bacterial type II fatty acid synthase [4]). This raises the possibility that BboHT1 has a novel architecture that may ultimately aid our understanding of the interaction between catechins and hexose transporters, serving as a negative control for gallated catechin binding.

Using a 3D structural model of GLUT1, Naftalin et al. [8] identified putative residues involved in the binding of EGCG (Asn 29, Thr 30, Arg 126, Ser 285, Asn 288, Asn 317 and Thr 318). Sequence alignment identified Arg 126 as the only residue conserved in GLUT1, PfHT and BboHT1. Additionally, the residue corresponding to Asn 317 in GLUT1 is conserved in PfHT, while the residues corresponding to Asn 29 and Thr 30 in GLUT1 are conserved in BboHT1. Given the different catechin susceptibility profile of BboHT1 compared with GLUT1 and PfHT, helix VIII sequences, which contain residues corresponding to Asn 317 in GLUT1, for a range of hexose transporters (mammalian, apicomplexan and kinetoplastidae) were aligned (Fig. 1A). In the case of Thr 318, there is little conservation between analogous residues. However, Asn 317 is highly conserved between transporters with the exception of the *Trypanosoma brucei* hexose transporter 1 (THT1), which also contains a polar residue at this position (Thr 351), and BboHT1, which is the only transporter aligned here that contains a hydrophobic residue, Leu 323, at this position.

To test (i) if the Asn residue in helix VIII of GLUT1 and PfHT is important for gallated catechin binding and (ii) if alteration of this residue can explain the altered pharmacology observed in BboHT1 (reported above), site-directed mutagenesis was used to introduce an Asn → Leu mutation in GLUT1 and PfHT and a Leu → Asn mutation in BboHT1. The effect of catechins on d-glucose uptake was then tested, using the mutant transporters expressed in *X. laevis* oocytes, and compared with data produced using the wild-type transporters (Fig. 1B–D).

In all cases, mutation reduced each transporter's assayed functionality (reducing uptake by at least 60%, when compared with wild-type controls, although these values are not corrected for protein expression), which made determination of catechin inhibition difficult and thus only the effect of a 0.5 mM concentration of each catechin was determined. Mueckler and Makepeace [13] reported that mutation of Asn 317 to a Cys residue in GLUT1 also reduced transport activity, where uptake data were normalised for transporter membrane expression. The mutant transporters preserved the same pharmacological catechin profile as wild-type transporters, with one exception: the mutant version of BboHT1 was affected significantly less (*P* < 0.001; ANOVA with Tukey's post-test) by EC when compared with wild-type BboHT1. The previously reported PfHT mutant Q169N [9] also had no effect on the action of catechins on d-glucose transport (data not shown).

The seven residues identified by Naftalin et al. [8] are predicted to reside on the rim of the external hexose binding site of GLUT1 (note that two of the residues, Thr 30 and Asn 317, were shown in mutagenesis experiments to be of significance in the GLUT1 exofacial binding site [14]), which would suggest a competitive mechanism of inhibition. Of these seven residues, three analogous residues in PfHT are conserved, corresponding to GLUT1 residues Arg 126, Asn 288 and Asn 317. However, mutation of the latter in GLUT1 and PfHT did not alter the gross effects of catechins on d-glucose transport. Although these mutations may have resulted in differential effects of gallated catechins at lower concentrations (experiments which were not performed because the mutations used here reduced measurable transporter activity), the data do not imply a primary role for Asn 317 and its analogous residues in EGCG binding.

Strobel et al. [15] produced a model for GLUT4 and performed docking studies with gallic acid, EC and ECG, with only the latter reaching a favourable energetic minimum. In the model, ECG was found possibly to interact with Gln 295 and Leu 296 of the QLS motif in transmembrane helix VII (a region which interacts with the C1 position of d-glucose and which is involved with substrate filtering in mammalian orthologues [16]). This suggests gallated catechins competitively inhibit d-glucose transport at the extracellular substrate binding site. However, while the data presented here do not rule out competitive inhibition, they are not consistent with a specific need for the interaction of gallated catechins with the QLS motif, as PfHT does not contain the motif but is inhibited by gallated catechins.

The surprising result described here is the reversed catechin pharmacological profile of BboHT1 compared with GLUT1, GLUT5 and PfHT. BboHT1 was also the only transporter that was significantly affected by mutation. In this case, the L323N mutation increased the amino acid conservation of BboHT1 to that of PfHT and GLUT1 and reduced the effect of EC (but not EGC), leaving the actions of ECG and EGCG unaltered. Further mutational studies in this region may help to elucidate the significance of this observation.

Table 1 shows IC_50_ values for the effect of ECG and EGCG on *P. falciparum* (3D7) growth *in vitro* (12 mM d-glucose) over 48 h, using methods described previously [11]. Furthermore, given that catechins are competitive inhibitors of d-glucose transport mediated by GLUT1 [8], the relationship between external d-glucose concentration and IC_50_ values was examined. For each catechin, altering the external d-glucose concentration from 12 mM down to 4 mM had no significant effect on parasite growth inhibition (*P* > 0.4; unpaired, two-tailed Student's *t*-test). The effect of catechins was also tested on *Babesia divergens* (strain Rouen 1987) growth as described [17]. At a concentration of 200 μM, EC, ECG, EGC and EGCG inhibited parasite growth by 66 ± 14, 91 ± 6, 30 ± 10, and 71 ± 2%, respectively (mean ± SEM; *n* = 3).

While gallated catechins were found to inhibit *P. falciparum* growth (with similar IC_50_ values to those reported previously [6,7]), changing the growth medium d-glucose concentration failed to alter the antimalarial effect of gallated catechins. If the inhibitory mechanism of ECG and EGCG is, indeed, competitive, this is not consistent with data reported for compound 3361 (a specific and competitive inhibitor of d-glucose transport via PfHT), which was used to validate PfHT as a drug target [11]. In addition, ECG was found to be the most effective inhibitor of *B. divergens* growth (killing over 90% of parasites at 200 μM) and, while it is not possible to state how closely the hexose transporter of *B. bovis* compares with that of *B. divergens*, it is worth noting that ECG was not a very effective inhibitor of BboHT1 and that compound 3361, a good inhibitor BboHT1, did not kill *B. bovis* parasites in culture [12]. Therefore, there is little information to suggest that the antimalarial effect of catechins results from the inhibition of hexose transporters.

In summary, we have shown that catechins are able to inhibit a diverse selection of phylogenetically distant facilitative hexose transporters and that, in keeping with previous reports, gallated catechins are more effective inhibitors than ungallated catechins. The exception to this rule is for catechin inhibition of BboHT1, a hexose transporter from *B. bovis* that is more susceptible to ungallated catechins. Bioinformatical and site-directed mutagenesis approaches provided little further evidence in support of previously proposed models of catechin binding with hexose transporters and, in the case of the QLS motif and Asn 317, ruled out primary roles. Finally, while catechins possess antimalarial activity, our data suggest that hexose transport is not their primary target.

## Figures and Tables

**Fig. 1:**
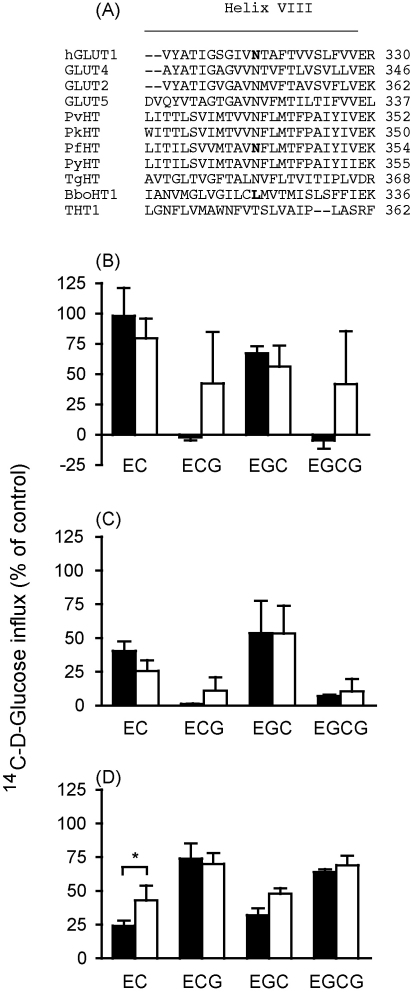
Sequence alignments of helix VIII from apicomplexan and mammalian hexose transporters and effect of catechins (0.5 mM) on hexose transport by wild-type (closed bars) and mutant hexose transporters (open bars). (A) The Clustal W program was used to generate the alignment. Bold residues were substituted in site-directed mutagenesis experiments. *Note*: NT are putative anchoring residues of GLUT1 with gallated catechins [8]. GLUT2, mammalian facilitated glucose transporter 2; GLUT4, mammalian facilitated glucose transporter 4; PvHT, *Plasmodium vivax* hexose transporter; PkHT, *P. knowlesi* hexose transporter; PyHT, *P. yoelii* hexose transporter, TgGT1, *Toxoplasma gondii* glucose transporter 1; THT1, *T. brucei* hexose transporter 1. (B) Wild-type PfHT versus N341L PfHT, (C) wild-type GLUT1 versus N317L GLUT1 and (D) wild-type BboHT1 versus L323N BboHT1. Uptake of [^14^C] d-glucose by PfHT, GLUT1 and BboHT1 was measured. All values were first corrected for the uptake into water-injected controls and are presented as a percentage of paired control experiments performed in the absence of any inhibitor. Data are averaged from three experiments, each on oocytes from a different toad, and are shown as means ± SEM. *Significantly different from wild-type (*P* < 0.001).

**Table 1:** Effect of catechins on PfHT, GLUT1, GLUT5 and BboHT1-mediated hexose transport (*K*_*i*_ values) and parasite growth (IC_50_ values).

Compound	Structure	*K*_*i*_ (μM)^a^				IC_50_ (μM)^a^
		PfHT	GLUT1	GLUT5	BboHT1	
EC		>500	∼500	>500	108 ± 52	nt
ECG		45 ± 10	68 ± 18	117 ± 54	>500	32 ± 1
EGC		>500	>500	>500	168 ± 83	nt
EGCG		90 ± 24	105 ± 18	113 ± 48	>500	17 ± 3

a Values are given as means ± SEM (*n* ≥ 3); nt, not tested.
